# Hybrid Model with Wavelet Decomposition and EfficientNet for Accurate Skin Cancer Classification

**DOI:** 10.7150/jca.101574

**Published:** 2025-01-01

**Authors:** Amina Aboulmira, Hamid Hrimech, Mohamed Lachgar, Mohamed Hanine, Carlos Osorio Garcia, Gerardo Mendez Mezquita, Imran Ashraf

**Affiliations:** 1 LAMSAD Laboratory, ENSA, Hassan First University, Berrechid, Morocco.; 2 L2IS Laboratory, FSTG, Cadi Ayyad University, Marrakech, Morocco.; 3 Higher Normal School, Department of Computer Science, Cadi Ayyad University, Marrakech, Morocco.; 4 LTI Laboratory, ENSA, Chouaib Doukkali University, El Jadida, Morocco.; 5 Universidad Europea del Atlantico, Isabel Torres 21, Santander, 39011, Spain.; 6 Universidad Internacional Iberoamericana, Campeche, 24560, Mexico.; 7 Universidade Internacional do Cuanza, Cuito, Angola.; 8 Department of Information and Communication Engineering, Yeungnam University, Gyeongsan, 38541, Republic of Korea.

**Keywords:** skin lesion, transfer learning, wavelet decomposition, image processing, convolutional neural networks

## Abstract

Faced with anomalies in medical images, Deep learning is facing major challenges in detecting, diagnosing, and classifying the various pathologies that can be treated via medical imaging. The main challenges encountered are mainly due to the imbalance and variability of the data, as well as its complexity. The detection and classification of skin diseases is one such challenge that researchers are trying to overcome, as these anomalies present great variability in terms of appearance, texture, color, and localization, which sometimes makes them difficult to identify accurately and quickly, particularly by doctors, or by the various Deep Learning techniques on offer. In this study, an innovative and robust hybrid architecture is unveiled, underscoring the symbiotic potential of wavelet decomposition in conjunction with EfficientNet models. This approach integrates wavelet transformations with an EfficientNet backbone and incorporates advanced data augmentation, loss function, and optimization strategies. The model tested on the publicly accessible HAM10000 and ISIC2017 datasets has achieved an accuracy rate of 94.7%, and 92.2% respectively.

## Introduction

Deep learning has had a huge impact on this century, resulting in considerable advances in many sectors 1. It has made a significant contribution and revolutionized many fields, including image recognition where convolutional neural networks (CNNs) have demonstrated their ability to automatically extract relevant features from images 2, often outperforming traditional methods. Also in natural language processing, where recurrent neural networks (RNNs) and transformers have achieved impressive results in various translation tasks and text generation 3, and computer vision where techniques such as suturing have been greatly improved thanks to neural networks 4.

Deep learning has opened up new opportunities in the realm of medicine, allowing for more accurate diagnosis of diseases from medical images, such as cancer detection, brain scan interpretation, and disease prediction from genetic data 5. It has also been employed in applications like health monitoring, medical records management, and drug discovery 6. In medical imaging, deep learning has additional challenges during the diagnosis of anomalies, such as the availability of a large amount of high-quality data annotated appropriately 7, which will undoubtedly contribute to the development of more efficient models. The imbalance between different classes of some diseases is present in various datasets, making it difficult for models to learn to detect abnormalities effectively 8. Models might be skewed toward the majority class, making it harder to detect uncommon cases correctly. The variability of data is also a real issue, which might make learning a generalizable model capable of detecting different anomalies in different contexts difficult 9.

Deep learning models, on the other hand, tend to learn from the special properties of the datasets on which they are trained. This can make it challenging to generalize and transfer learned information to new datasets or anomalies 10. Model generalization and transfer can be improved using techniques such as knowledge transfer, domain learning, and unsupervised learning 11.

Skin disorders are frequent conditions that can develop for a variety of reasons and have many causes. Many skin disorders, including psoriasis, eczema, and various kinds of dermatitis, are influenced by genetic factors. Allergic reactions to things such as chemicals, medications, cosmetics, or allergens in the environment are other common causes of skin illness. Another prevalent cause of skin illness includes bacterial, viral, or fungal infections, which can lead to conditions such as acne, warts, herpes, ringworm, or candidiasis, among other skin problems 12. One emerging challenge is the rapid evolution of skin disease presentations, particularly due to environmental changes and evolving pathogen strains 13. Climate change and pollution are contributing to new dermatological conditions and altering the presentation of existing ones, making it more difficult for both clinicians and diagnostic models to keep up with these changes 14.

Furthermore, the integration of digital health technologies in dermatology has led to the development and widespread adoption of computer-aided diagnosis (CAD) systems. These systems have the potential to significantly enhance the accuracy and efficiency of diagnosing skin diseases. By leveraging advanced algorithms and machine learning models, CAD systems can assist clinicians in quickly identifying both common and rare skin conditions, facilitating faster decision-making in clinical settings. This rapid diagnostic capability is particularly beneficial in busy healthcare environments, where early and accurate detection is crucial for effective treatment and improved patient outcomes. CAD systems can serve as valuable tools, especially for less experienced practitioners, by providing reliable second opinions and reducing the likelihood of diagnostic errors 15.

However, the effective use of CAD systems is not without its challenges. Skin disorders can exhibit a wide range of clinical symptoms, making exact diagnosis challenging 16. The same skin conditions might manifest differently in various individuals, necessitating a thorough understanding of each disease's distinctive characteristics 17. Moreover, the similarity between many skin conditions can further complicate the diagnostic process, even for advanced CAD systems. Ensuring that these systems can accurately distinguish between similar conditions remains a critical area of focus in dermatology 18. Many skin diseases might have similar symptoms and characteristics, making precise differentiation difficult. Even experienced dermatologists may face difficulties distinguishing certain diseases based on visual observations 19. These difficulties highlight the importance of a rigorous approach to diagnosing skin diseases using deep learning. Continuous research efforts are required to overcome these challenges, improve the quality of data sets, and develop deep learning models that are precise, interpretable, and capable of generalizing to different populations and environments 20.

Indeed, there have been many contributions to using deep learning in the diagnosis of skin diseases 21. These approaches have leveraged various deep learning techniques, such as the ability of networks to extract complex features from images and perform accurate classifications. Models trained on large databases of dermatological images can identify specific patterns, such as lesions, spots, or textures, enabling precise classification of skin diseases 22. For example, a multistage multiclass CNN-based framework has set a benchmark with an accuracy of 0.96 23, demonstrating the potential of deep learning models in this domain. However, this method employs a two-stage classification process, which, while effective, introduces additional complexity that could increase computational costs and limit scalability in practical applications.

In contrast, the proposed approach integrating wavelet features into a CNN achieved a competitive accuracy of 0.947. Although slightly lower than the benchmark, this method simplifies the classification process by directly enhancing the CNN with wavelet features, thereby avoiding the need for a multi-stage approach. This not only reduces computational complexity but also offers distinct advantages in capturing both spatial and frequency domain information, potentially leading to better generalization and robustness in clinical settings. Unlike methods such as the Ant Colony and Whale Optimization Algorithms 24, which focus on optimizing neural networks, or the integration of handcrafted and deep learning features, this approach systematically combines wavelet features with CNNs to improve classification performance in challenging cases. Additionally, while Vision Transformers have gained attention for modeling global dependencies, this method emphasizes capturing both local and multi-scale information, providing a complementary approach to these recent advances.

This study introduces a novel hybrid architecture combining wavelet decomposition with EfficientNet models for skin cancer classification. Wavelets are mathematical functions used to analyze images by decomposing the data into different scales and frequencies 25. This approach enables significant information to be extracted at different spatial and frequency resolutions. By integrating wavelet features with EfficientNet, the model leverages the powerful representational capabilities of EfficientNet while enhancing the ability to capture fine-grained details in skin lesions. This hybrid model aims to strike a balance between complexity and performance, ensuring that high accuracy is maintained without the need for overly complex processing pipelines. The extracted wavelet coefficients can be used as input for layers of the EfficientNet neural network, known for its robustness 26, to improve the model's discrimination capability, as wavelets can be used to analyze the textural and structural characteristics of dermatological images. This hybrid approach leverages the strength of EfficientNet and enhances it with the wavelet transform's ability to decompose images into frequency sub-bands, capturing both spatial and frequency information. The main contributions of this paper are:

- Analyzing the effect of the Wavelet decomposition on skin disease images.
- Conception of a novel hybrid model based on Wavelets and CNN Block.
- Development of a hybrid version of the EfficientNet model based on a fusion of Wavelets and CNN blocks.

The next section goes over the background of the Wavelet transform and convolutions. Following that the related work is discussed. The methodology is then presented, along with the public skin disease datasets description, the preprocessing stage, and the conception of the Efficientnet combined with the Wavelet decomposition process. Then the experimental results are presented and analyzed, along with discussion of the limitations of the proposed methodology and potential directions for future research, and lastly the conclusion.

### Background

This section looks at the essentials of modern deep learning research, providing a comprehensive overview of EfficientNet, and wavelet decomposition. EfficientNet, a state-of-the-art convolutional neural network, optimizes both network depth and width for consistently superior performance. Wavelet decomposition is emerging as a powerful tool for multi-resolution analysis, enabling more efficient representation and processing of signals and images. Building on these fundamental techniques, the combination of a CNN network integrating wavelet transforms with CNNs enhances feature extraction and representation capabilities.

### EfficientNet

The EfficientNet architecture has been a game-changer in the world of deep learning, especially for tasks like identifying objects in images. It was developed by Tan and Le back in 2019 27. One of its main versions, EfficientNetB0, has gained a lot of attention for its ability to perform well and use resources efficiently when classifying images. It is renowned for its compound scaling method, which basically tweaks the model's size and complexity to find the perfect balance. This means it can deliver top-notch accuracy without consuming too much computing power, making it suitable for AI models integrated with devices. In this study, the initialization of the EfficientNet model was done with noisy student weights. This helps improve the model's performance and adaptability to the target task.

### Wavelet decomposition

**Figure 1:**
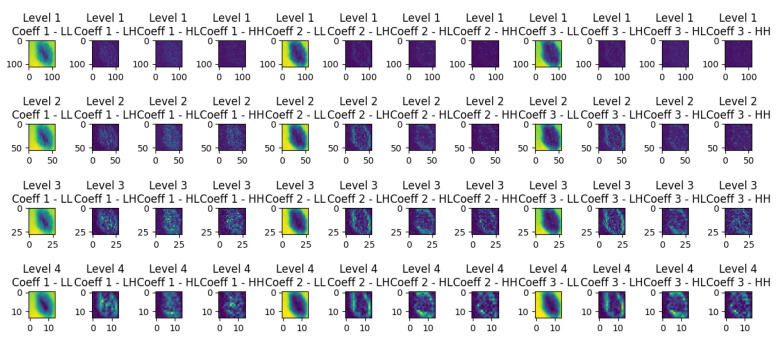
Wavelet decomposition levels of a dermoscopic image.

Wavelet decomposition is a technique used to analyze and represent the spatial frequency content of an image at different scales and orientations. In Figure 1, resulting from wavelet decomposition, each level corresponds to a different scale of features within the original image. The **LL** (low-low) coefficients capture the coarse details or low-frequency information, resembling a downsampled version of the original image. Meanwhile, the **LH** (low-high), **HL** (high-low), and **HH** (high-high) coefficients encode finer details, horizontal edges, vertical edges, and diagonal features, respectively. As one progresses through the decomposition levels, the images reveal increasingly detailed and localized information:

- **Level 1 Decomposition:** provides a coarse approximation and detailed coefficients that capture high-frequency components along both axes and diagonals. This level is closest to the original image size, retaining most of the spatial information but with the initial layer of abstraction.
- **Level 2 Decomposition:** goes deeper, offering a finer analysis. It starts to distill the image into more abstract representations, emphasizing features that might be less obvious in the raw pixels. This level balances spatial resolution and feature abstraction, making it suitable for tasks where both global and local features are important.
- **Level 3 and Level 4 Decomposition:** offer increasingly abstract representations of the image data. These levels focus more on the high-level features that might be relevant for understanding complex patterns or for tasks where the exact spatial location of features is less critical than the features' presence.

Wavelets can be used to examine the textural and structural properties of dermatological images in the field of skin disease diagnostics. Wavelet analysis, for example, can capture skin texture, patterns, contours, and unique properties associated with specific skin disorders. The derived wavelet coefficients can be utilized to represent features of skin disease images as descriptors 25. These descriptors can be used to build classification models or to pinpoint specific areas of interest in a picture. The use of wavelets can help reduce data dimensionality, which is useful for processing and analyzing big dermatological picture databases.

### Wavelet decomposition algorithm

Wavelet decomposition is used to enhance the feature extraction process from image batches, enabling improved classification effectiveness. The process of wavelet decomposition of image data into multiple sets of coefficients that represent different frequency components is detailed as follows:

Require: Image batch IEnsure: Decomposed coefficients at multiple levels

function WaveletDecomposition (I)Initialize empty list coefficients ⊳ Level 1 DecompositionI ← permute(I, [0, 3, 1, 2])R, G, B ← I[:, 0], I[:, 1], I[:, 2]

(R_L, R_H) ← WaveletTransformAxisY(R)(R_LL, R_LH) ← WaveletTransformAxisX(R_L)(R_HL, R_HH) ← WaveletTransformAxisX(R_H)(G_L, G_H) ← WaveletTransformAxisY(G)(G_LL, G_LH) ← WaveletTransformAxisX(G_L)(G_HL, G_HH) ← WaveletTransformAxisX(G_H)(B_L, B_H) ← WaveletTransformAxisY(B)(B_LL, B_LH) ← WaveletTransformAxisX(B_L)(B_HL, B_HH) ← WaveletTransformAxisX(B_H)

wavelet_data ← [R_LL, R_LH, R_HL, R_HH, G_LL, G_LH, G_HL, G_HH, B_LL, B_LH, B_HL, B_HH]transform_batch ← stack(wavelet_data, axis = 1) ⊳ Multilevel Decomposition

for k = 2 to … do(L^(k), H^(k)) ← WaveletTransformAxisY(L^(k-1))(LL^(k), LH^(k)) ← WaveletTransformAxisX(L^(k))(HL^(k), HH^(k)) ← WaveletTransformAxisX(H^(k))Append (L^(k), H^(k), LL^(k), LH^(k), HL^(k), HH^(k)) to coefficientsend for

Permute decomposed components back to original dimensionsdecom_level_1 ← permute(transform_batch, [0, 2, 3, 1])decom_level_2 ← permute(transform_batch_l2, [0, 2, 3, 1])decom_level_3 ← permute(transform_batch_l3, [0, 2, 3, 1])decom_level_4 ← permute(transform_batch_l4, [0, 2, 3, 1])

return decom_level_1, decom_level_2, decom_level_3, decom_level_4end function

function WaveletTransformAxisY (I)I_odd ← I[:, 0::2]I_even ← I[:, 1::2]L_y ← (I_odd + I_even)/2H_y ← |I_odd - I_even|return L_y, H_yend function

function WaveletTransformAxisX (I)I_tmp ← permute(I, [0, 2, 1])[:, :, ::-1]L_x, H_x ← WaveletTransformAxisY(I_tmp)L_x ← permute(L_x, [0, 2, 1])[:, ::-1, …]H_x ← permute(H_x, [0, 2, 1])[:, ::-1, …]return L_x, H_xend function

### Residual wavelet network

**Figure 2:**
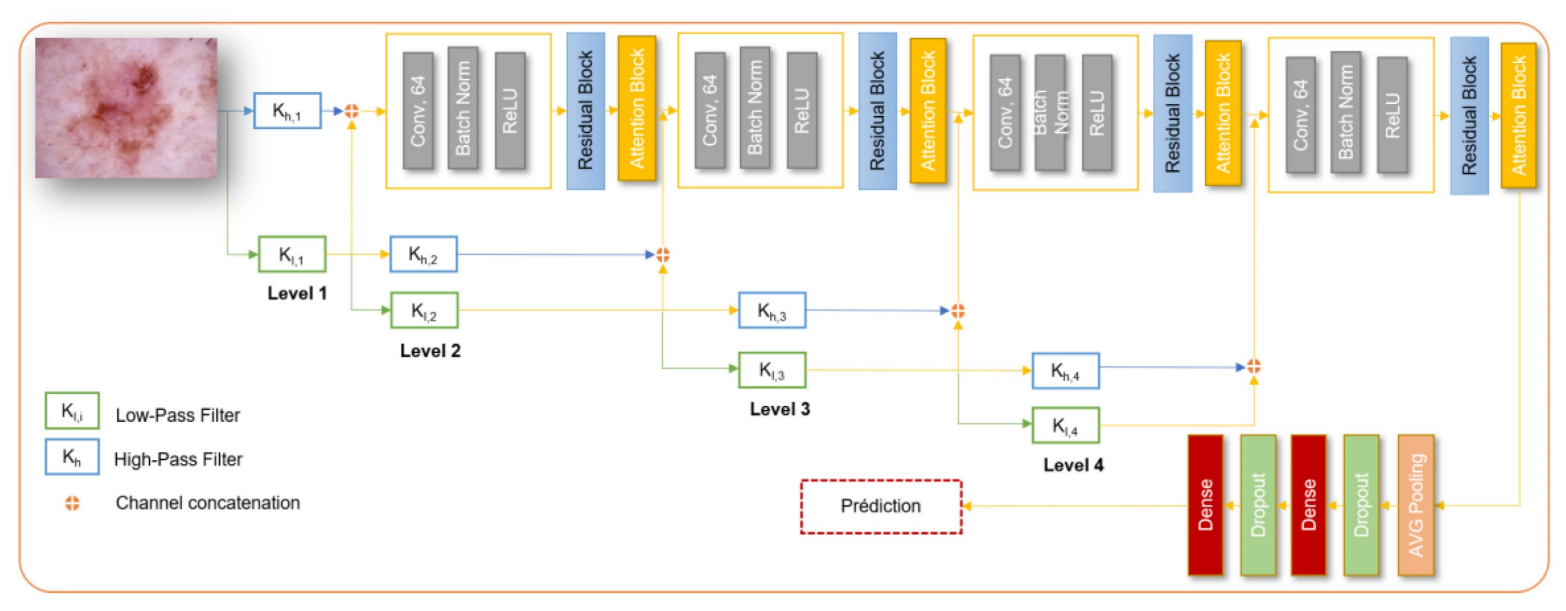
Naive Wavelet convolutional neural network.

The architecture begins with preprocessing the input dermoscopic images using wavelet transformations to capture frequency-specific features. The wavelet coefficients are then adjusted to enhance their representational capacity. The subsequent structure involves convolutional layers with Relu activation, max pooling, and residual blocks, which facilitate the extraction of hierarchical features from the images. The inclusion of residual blocks enables the network to learn and represent complex patterns effectively. Figure 2 depicts the overall architecture of the model.

The architecture utilizes four levels of wavelet-decomposed inputs, each processed independently through corresponding residual blocks. Additionally, the features extracted from different levels of the wavelet decomposition are fused with the features from the residual blocks. This fusion process enhances the model's ability to capture diverse and complementary information from the input images. The final output layer performs multi-class classification, categorizing the input images into different skin disease classes. This network is considered as a Naive implementation of a CNN network with wavelet.

### Related work

Image classification using wavelet techniques involves utilizing wavelet transforms to extract features from images that can then be used for classification purposes. Wavelet transforms are mathematical functions that analyze signals or images by decomposing them into different frequency components. This approach can enhance the ability of a model to capture both local and global features of an image, making it well-suited for image classification tasks. Wavelets have also been explored in the context of skin disease diagnosis. Hybrid approaches have been proposed to improve skin disease diagnosis 28, 29, 30.

In 31, Indira et al. proposed a novel approach for enhancing skin cancer detection and analysis through a Texture Analysis-based Classification Module. The method employs key symptoms of skin cancer, including Asymmetry, Border Irregularity, Color variation, and Diameter, in the processing algorithm. By utilizing multi-level Wavelet Transformation on input images and selecting specific sub-bands for optimal defect detection, the proposed system aims to improve decision accuracy in skin cancer detection and analysis, offering a promising avenue for more effective diagnostic strategies. However, the method primarily focuses on classification based on texture analysis and multi-level wavelet transformation, which may not fully leverage the potential of modern deep learning architectures for feature extraction and classification.

Alenezi et al. in 28 proposed an innovative approach for accurate skin lesion classification using a wavelet transform-based deep residual neural network (WT-DRNNet) combined with the ReLU-based Extreme Learning Machine (ELM). The model integrates wavelet transformation, pooling, and normalization to enhance image details while removing unwanted artifacts from skin lesion images. Deep features are extracted using a residual neural network through transfer learning, and these features are then combined with global average pooling. The model is evaluated using the ISIC2017 and HAM10000 datasets, achieving impressive results: on the ISIC2017 dataset, metrics such as accuracy, specificity, precision, and F1-Score reached 96.91%, 97.68%, 96.43%, and 95.79% respectively, and 95.73%, 98.8%, 95.84%, and 93.44% respectively, on the HAM10000 dataset, outperforming existing methods. While achieving impressive accuracy, the integration of multiple steps (wavelet transform, feature extraction, neural network processing, and ELM) introduces complexities and increases execution time for testing. This higher computational demand may not be feasible in all deployment environments due to limited resources.

Serte et al. in 29 presented two novel methods for the automatic classification of malignant melanoma and seborrhoeic keratosis skin lesions. The first method leverages wavelet coefficients obtained through wavelet transformation, combined with deep learning models for skin image representation. The second method employs sequential wavelet transformation to produce approximation coefficients, followed by deep learning model application. Using transfer learning-based ResNet-18 and ResNet-50 models, both image and coefficient representations are analyzed, and model output probabilities are fused for lesion detection. Comparative results highlight the superiority of the proposed approach, with ResNet-18-based I-A1-H-V and ResNet-50-based I-A1-A2-A3 models achieving remarkable M-AUC value of 96% and M-ACC value of 85% on ISIC2017 dataset, effectively surpassing other recent methods for melanoma detection. The approach involves two separate methods combining wavelet coefficients with deep learning models, but the integration is not as seamless. The sequential wavelet transformation and separate deep learning application might result in complexity and potential overfitting.

In 30, Chatterjee et al. presented a systematic methodology for computer-aided identification of four classes of skin diseases. By employing empirical wavelet transform, dermoscopic images are decomposed into various frequency spectra to analyze complex textural properties of skin lesions. The empirical wavelet fractal descriptor (EWFD) is introduced for quantitative textural complexity analysis. Morphological, texture, and color features are extracted, and a recursive feature elimination-based technique is used for feature selection. Employing ensemble multiclass classification, the proposed approach achieves high sensitivities of 99.20%, 98.60%, 98.20%, and 98.80% for melanoma, nevus, BCC, and SK diseases, respectively. The study's conclusion highlights the successful identification of similar skin abnormalities, with empirical wavelet transform and the introduced EWFD proving effective for quantifying textural complexity. The ensemble multiclass classification demonstrates the potential of this method for accurate and comprehensive skin disease identification. While effective, the approach may be computationally intensive due to the recursive feature elimination and ensemble techniques.

Aboulmira et al. in 32 provides a significant advancement in the field of dermatological image classification by leveraging Fast Fourier Transform (FFT) convolution layers. This study focuses on integrating FFT-based convolution with a denoising block within a ResNet-18 architecture, aiming to enhance the efficiency and accuracy of skin disease classification. Utilizing the HAM10000 dataset, the research achieved an accuracy rate of 88%, highlighting the method's superiority compared to traditional convolution techniques. The authors suggest that future research should explore the use of larger kernels and the potential of frequency domain learning to further improve classification outcomes. Unlike FFT-based methods, the use of wavelet decomposition in this study allows for a multi-resolution analysis of images, capturing both frequency and location information more effectively.

Wavelet transformation enables multi-resolution analysis, which enhances the model's ability to capture both fine and coarse features in skin images. This is particularly beneficial for dermatological applications, where lesions and skin abnormalities can vary significantly in size and texture. While previous studies have effectively utilized wavelets, the proposed hybrid architecture addresses existing research gaps by directly integrating wavelet transformations with the EfficientNet backbone in specific layers. This results in a unified, robust combined model. Second, the model isolates specific frequencies and leverages pretrained Efficientnet features for enhanced detection avoiding redundant processing. This hybrid approach not only improves the model's robustness and precision but also facilitates the extraction of more meaningful features, leading to better diagnostic capabilities. Furthermore, the combination of these techniques can potentially reduce the model's susceptibility to overfitting, making it more reliable for clinical applications. As a result, this hybrid model represents a significant advancement in the automated diagnosis of skin diseases, promising enhanced diagnostic performance and greater generalizability across diverse datasets.

## Methodology

This section outlines the methodology employed, detailing the workflow, dataset, data augmentation, preprocessing, and model training process. The workflow begins with the acquisition and preparation of the dataset, followed by extensive data augmentation to enhance the model's generalization capabilities. The training process leverages EfficientNet architecture, integrating wavelet transforms to refine feature extraction and improve performance.

### Workflow

The core of the proposed methodology is a hybrid CNN architecture that combines the high-level feature extraction capabilities of EfficientNet with the multi-resolution analysis strengths of wavelet transforms. The model architecture integrates wavelet-transformed inputs into an EfficientNet backbone at strategic insertion points, allowing for the capture of both spatial and frequency domain features pertinent to skin lesion classification. The wavelet transformation layer decomposes input images into frequency subbands using a discrete wavelet transform, effectively capturing intricate texture details. These wavelet-transformed features, weighted by learnable parameters, are then integrated with the corresponding EfficientNet feature maps through an Add layer, resulting in a unified feature representation. Towards the end of the model, global average pooling and dropout layers are utilized to perform dimensionality reduction and provide regularization, ensuring robust and efficient learning.

**Figure 3:**
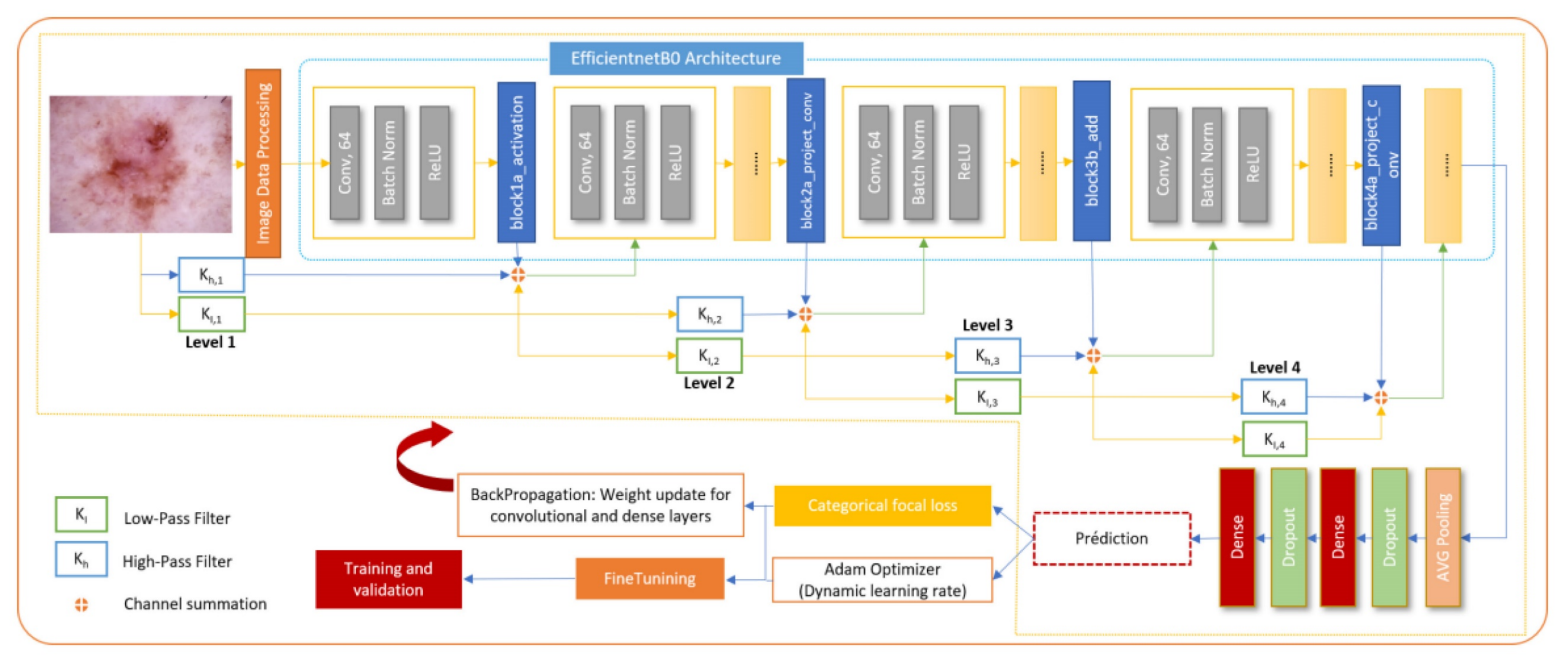
General workflow of the proposed hybrid model.

The proposed architecture strategically integrates wavelet-transformed inputs into the EfficientNet backbone at carefully selected insertion points, specifically after the block1a_activation, block2b_add, and block4a_project_conv layers. Early insertion at block1a_activation enhances low-level feature extraction with additional frequency information, mid-layer insertion at block2a_project_conv complements mid-level patterns, and late insertion at block4a_project_conv enriches high-level, abstract features. This strategic placement ensures that wavelet-transformed data contributes effectively to the model's overall feature representation, optimizing the balance between spatial and frequency-domain information while maintaining computational efficiency and model accuracy. Figure 3 depicts the overall process of classification of skin diseases.

To enhance the training dynamics, a warmup phase is introduced at the beginning of the training process. During this phase, the learning rate is gradually increased, allowing the model to transition from its initial random initialization to more stable learning. Following the warmup, a cosine learning rate scheduler is employed. This scheduler adjusts the learning rate as training progresses, contributing to effective convergence and optimized model performance. Furthermore, learning rate adjustments are incorporated through callbacks. Callbacks are functions that influence the training process at various stages. By dynamically adjusting the learning rate during training, the network's ability to adapt and learn from the data is finetuned. This aspect demonstrates a sophisticated understanding of neural network training strategies, aiming to strike a balance between exploration and exploitation in the learning process.

Overall, this model offers interesting features for skin disease diagnosis. Combining EfficientNet features with Wavelet decomposition presents a promising solution to enhance classification tasks by leveraging the strengths of both the EfficientNet and wavelet network.

### Dataset

In this study, two well-established datasets, HAM10000 and ISIC2017, were utilized, serving as critical benchmarks in the domain of skin disease classification. A detailed description of each dataset is provided below:

- HAM10000 (Human Against Machine): is a diverse dataset which contains 10015 dermoscopy images representing skin lesions across seven diagnostic categories: actinic keratosis (AKIEC), basal cell carcinoma (BCC), benign keratosis (BKL), dermatofibroma (DF), melanoma (Mel), melanocytic nevus (NV), and vascular lesions (VASC) 33. The dataset is available at: https://www.kaggle.com/datasets/artakusuma/basedir.
- ISIC 2017: Part of the International Skin Imaging Collaboration (ISIC) challenge, consisting of 2,000 images with annotations for melanoma diagnosis 34. The dataset is accessible via: https://www.kaggle.com/datasets/johnchfr/isic-2017.

In this experiment, the datasets were randomly divided into a training, validation, and test set a 70:20:10.

### Data augmentation

Data augmentation aims to increase the amount of data by performing transformations on the data already existing in the dataset 35. Different transformations were used, such as random horizontal and vertical Flips, random Rotation, and color normalization.

Indeed, the distribution of color values varied according to lighting conditions, cameras, and other factors, as noted by Mbatha et al. 36 has a negative impact on the results and is therefore characterized as noise. For this reason, a color normalization phase was applied by averaging and normalizing three channels over the entire dataset. The 'Shades-of-Grey' algorithm 37 was also applied, which normalizes the color in images to mitigate the variance introduced by different imaging conditions and devices 38.

### Fine tuning

The process of finetuning involves initially locking all layers of the EfficientNet model to keep their pre-trained weights unchanged during the early stages of training. As training progresses, layers are progressively unlocked, beginning with those nearest to the output, to fine-tune the model's pre-existing features for a particular task. In this specific approach, the first step involved unlocking the top 10 layers for adjustment, followed by the top 20, and eventually all layers were made adjustable.

This approach allows the model to first adjust the newly added layers to the task at hand without disturbing the pre-trained features. Gradually unfreezing more layers lets the model start fine-tuning the more abstract representations in the pre-trained model to better suit your specific dataset and task.

## Experimental Results and Discussion

### Set up for experiments

For performance evaluation, the proposed architecture was tested on two common dermatological datasets: HAM10000 and ISIC2017. The method is implemented in Tensorflow, and the experiments were done with GeForce RTX 2060 GPU - 6GB GDDR5 memory and memory (RAM) of 16.0GB on a Windows 10 machine. The training of the model is meticulously designed to optimize performance. The categorical focal loss function is selected as the loss function to address class imbalance within the dataset. The Adam optimizer is utilized for its adaptive learning rate capabilities, facilitating efficient convergence. A dynamic learning rate scheduler is implemented to adjust the learning rate based on training progress, reducing the learning rate when the validation loss plateaus to refine the model's accuracy and prevent overfitting.

The model is trained with a batch size of 16, which balances computational efficiency and model performance, and training is conducted until convergence, monitored by early stopping criteria based on validation loss to prevent overtraining. The evaluation focused on accuracy, precision, recall, and F1-score as primary metrics to assess the model's performance across three scenarios: using the EfficientNet backbone without wavelet decomposition (Baseline), Naive CNN wavelet decomposition, and the combined approach where wavelet features are integrated into the EfficientNet architecture gradually. The table 2 presents the results of an ablation study that evaluates the impact of different wavelet decompositions on the performance of the EfficientNet model. Metrics including accuracy, precision, recall, and F1-score are reported for various configurations, demonstrating the contribution of each wavelet decomposition level to the overall model performance.

It is evident that the proposed model achieves a greater accuracy compared to the baseline EfficientNet model. The Naive Wavelet-CNN model is the baseline model, and it achieves a reasonable accuracy. The precision, recall, and F1 scores are also fairly balanced. The time per step is relatively fast. This suggests that combining advanced wavelet techniques with CNN layers can significantly improve diagnostic accuracy in medical imaging.

Using the EfficientNetB0 architecture along with noisy training has resulted in a substantial accuracy improvement. The precision, recall, and F1-score are all at a high level, showing the model's effectiveness. The time per step is relatively moderate. The use of wavelet-transformed features through a few layers of EfficientNet has significantly improved accuracy and F1-score compared to the baseline model. The precision and recall are also high, indicating that the model performs well on both positive and negative samples. The time per step is slightly slower, likely due to the combination process, but still reasonable. This indicates that combining predictions from wavelet decomposition in general contributes positively to performance. However, it can be noticed that Efficientnet features integration with the first level of wavelet decomposition decreases the performance compared to using only the baseline EfficientNet, this can be due to the fact that Efficientnet's early layers capture basic and general features like edges and textures. Integrating wavelet features here can have a risk of overwhelming the model with too much detail too early, especially if the wavelet features are complex.

Integrating the second level of wavelet decomposition leads to the most significant performance improvement among the other levels of fusions. This can be due to the nature of the middle layers of EfficientNet which have begun to abstract away from the most basic features but are still flexible in terms of adapting to additional information. Integrating wavelet features at this stage can enrich the model's feature set as they preserve more spatial information, which can complement EfficientNet's capability to extract detailed features. Level 2 decomposition provides a good balance between retaining important spatial information and abstracting away details to highlight features that may be more subtly indicative of specific skin conditions, making the model suitable for capturing the unique aspects of different skin diseases.

For the third and fourth wavelet decomposition, it can be seen that there's no great impact on performance, the reason can be that the deeper layers of EfficientNet are highly abstract and task-specific. Adding wavelet features at this stage could potentially dilute the specificity of the network's learned representations if the added features are not sufficiently aligned with the task. Fusion of all the levels of wavelet decomposition introduces additional parameters and complexity into the model. While this can be beneficial for integrating diverse information sources, it hinders the model's ability to learn effectively. This can be due to redundancy without adding new information, potentially confusing the model or diluting important signals with repeated data, which leads to overfitting.

Class imbalance is a significant challenge in medical image datasets, such as HAM10000, where certain skin conditions are much more prevalent than others. This imbalance can lead to models that perform well on majority classes but poorly on minority classes, resulting in inflated accuracy and potential overfitting.

To address this issue, categorical focal loss was employed, which specifically targets class imbalance by down-weighting the loss contribution of well-classified examples and focusing more on hard-to-classify cases, often belonging to minority classes. Additionally, data augmentation techniques, such as rotation, flipping, and color normalization, were applied to increase the representation of minority classes in the training data. Table 3 presents the per-class performance metrics on the HAM10000 dataset for the three different methods: Naive Wavelet, Baseline EfficientNetB0, and Fusion with 2nd Level Wavelet Decomposition, across the seven types of skin lesions: AKIEC, BCC, BKL, DF, MEL, NV, and VASC. Performance is measured using the F1-score, a harmonic mean of precision and recall, which provides a balance between the model's accuracy and its ability to detect positive samples.

The Baseline EfficientNetB0 generally improves upon the Naive Wavelet method, especially notable in AKIEC (0.64), BCC (0.82), and BKL (0.77), reflecting the strength of EfficientNetB0 in capturing more complex features compared to the Naive Wavelet method. Despite these improvements, it shows only marginal progress or even a slight decline in categories like VASC, indicating that the method might not uniformly enhance detection across all conditions.

Naive Wavelet CNN shows varied performance across the categories, with its highest F1-score in NV (0.97) and VASC (0.85), indicating strong performance in these particular conditions. However, it struggles with MEL (0.45) and AKIEC (0.50), suggesting limitations in identifying these conditions accurately. The Fusion with 2nd Level Wavelet Decomposition approach demonstrates superior performance across all categories, with the most significant improvements observed in DF (0.91), MEL (0.77), and AKIEC (0.78).

**Figure 4:**
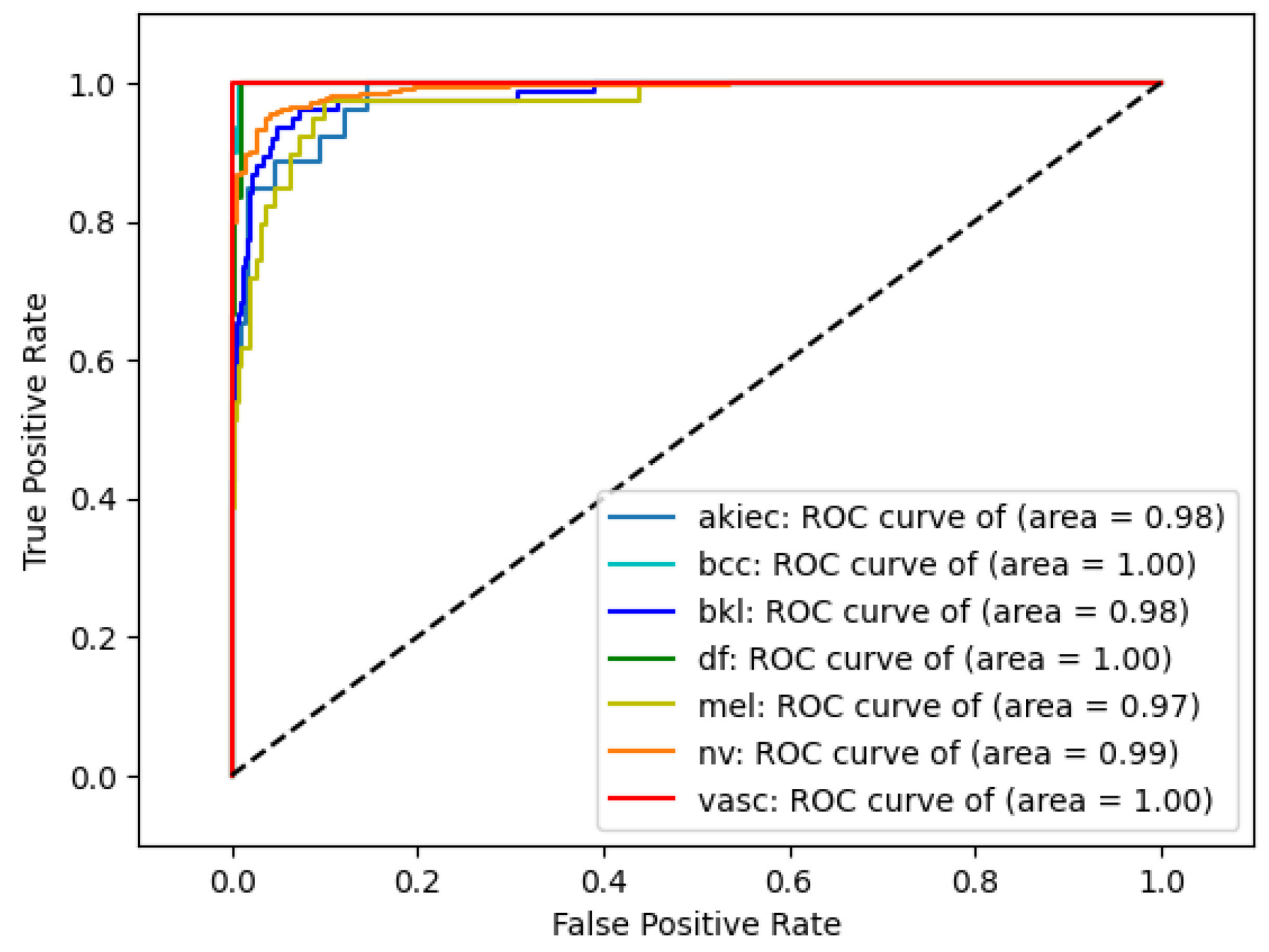
ROC Curve for Wave-EfficientNet Model.

**Figure 5:**
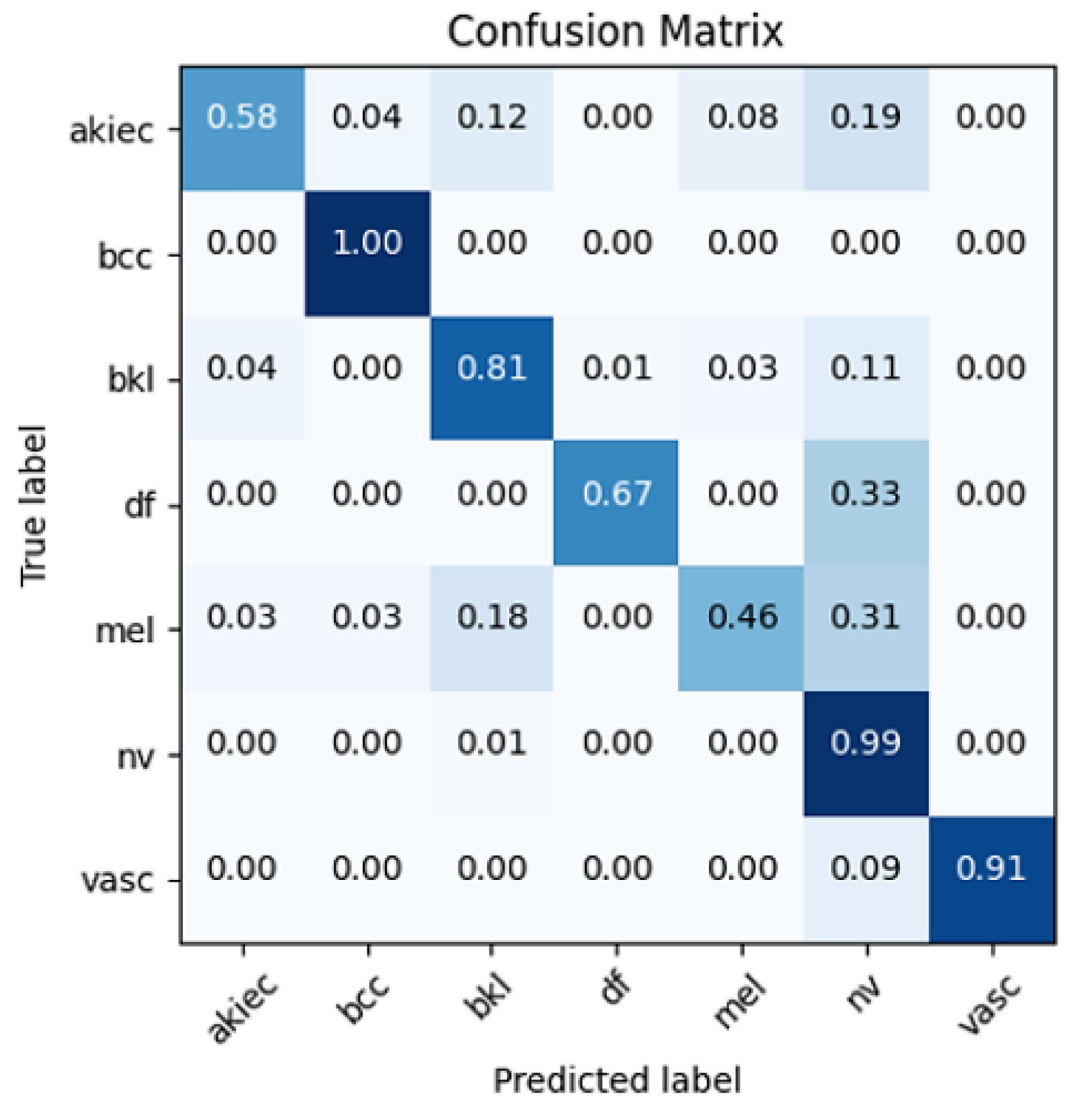
Matrix confusion of the best model performance.

This suggests that integrating 2nd-level wavelet decomposition with EfficientNetB0 significantly enhances the model's ability to detect a wide range of conditions, likely due to the method's effectiveness in capturing both high-level and detailed features within the data. These improvements are further corroborated by the confusion matrix (Figure 5) and ROC curves (Figure 4), which demonstrate that the model has learned to distinguish between different classes effectively, without overfitting to the majority class, NV.

Despite these mitigation strategies, some classes remain more challenging, as reflected in slightly lower F1-scores for classes like MEL and AKIEC. Future work could explore additional techniques, such as adaptive resampling or cost-sensitive learning, to further enhance performance on these minority classes.

It is imperative to evaluate not just accuracy but also precision, recall, F1-score, and ROC score, particularly given the significant imbalance within the dataset. Table 4 showcases the performance of an EfficientNet model with second wavelet decomposition (Wave-Efficientnet), highlighting its effectiveness through three metrics: Micro ROC score (0.996), Macro ROC score (0.988), and Accuracy (0.947). These scores reflect the model's ability to accurately classify instances, demonstrating its robustness across both individual predictions and various classes. The ROC curve further in Figure 4 illustrates the model's discriminative power, providing a comprehensive visualization of its performance across different thresholds.

To provide a comprehensive view of the classification outcomes from the most effective model, in terms of correct and incorrect classifications, confusion matrices are included in Figure 5.

A series of ablation studies were conducted to evaluate the effectiveness of the proposed hybrid architecture. These studies focus on different fusion techniques and structural modifications within the model, aiming to understand the contributions of each component to the overall performance. Specifically, the investigations covered:

1. The effect of direct addition versus concatenation of wavelet features to the EfficientNet backbone.

2. The efficiency and performance implications of modifying the insertion point of wavelet features within the EfficientNet model.

3. The impact of employing various fusion strategies, including simple summation, weighted summation, attention-based fusion, and concatenation followed by 1x1 convolution.

The results of the ablation study are summarized in Table 5, where each configuration's performance is evaluated in terms of accuracy, precision, recall, F1-score, and inference time. This comprehensive comparison identifies the most effective architectural modifications for optimizing the hybrid model's performance.

The results in Table 5, indicate that early wavelet feature insertion generally yields superior performance across all evaluated metrics, with the configuration using direct addition via an Add layer with weighted summation achieving the highest overall performance. This method produced an accuracy of 94.7%, a precision of 95%, a recall of 94%, and an F1-score of 94%. The Add layer without weighted summation approach yielded similar results, suggesting that seven without explicit weighting, the early fusion of wavelet features with the use of the Add layer, contributes significantly to the overall classification accuracy and generalization capability of the model.

In contrast, late wavelet feature fusion, particularly using concatenation followed by 1x1 convolution, resulted in a notable decline in performance. The complexity introduced by this method appears to detract from the model's ability to generalize, as evidenced by the lower accuracy (91.79%) and F1-score (92%). These findings suggest that early integration of wavelet features allows the network to more effectively leverage the complementary information provided by the wavelet decompositions, leading to improved classification performance.

**Figure 6:**
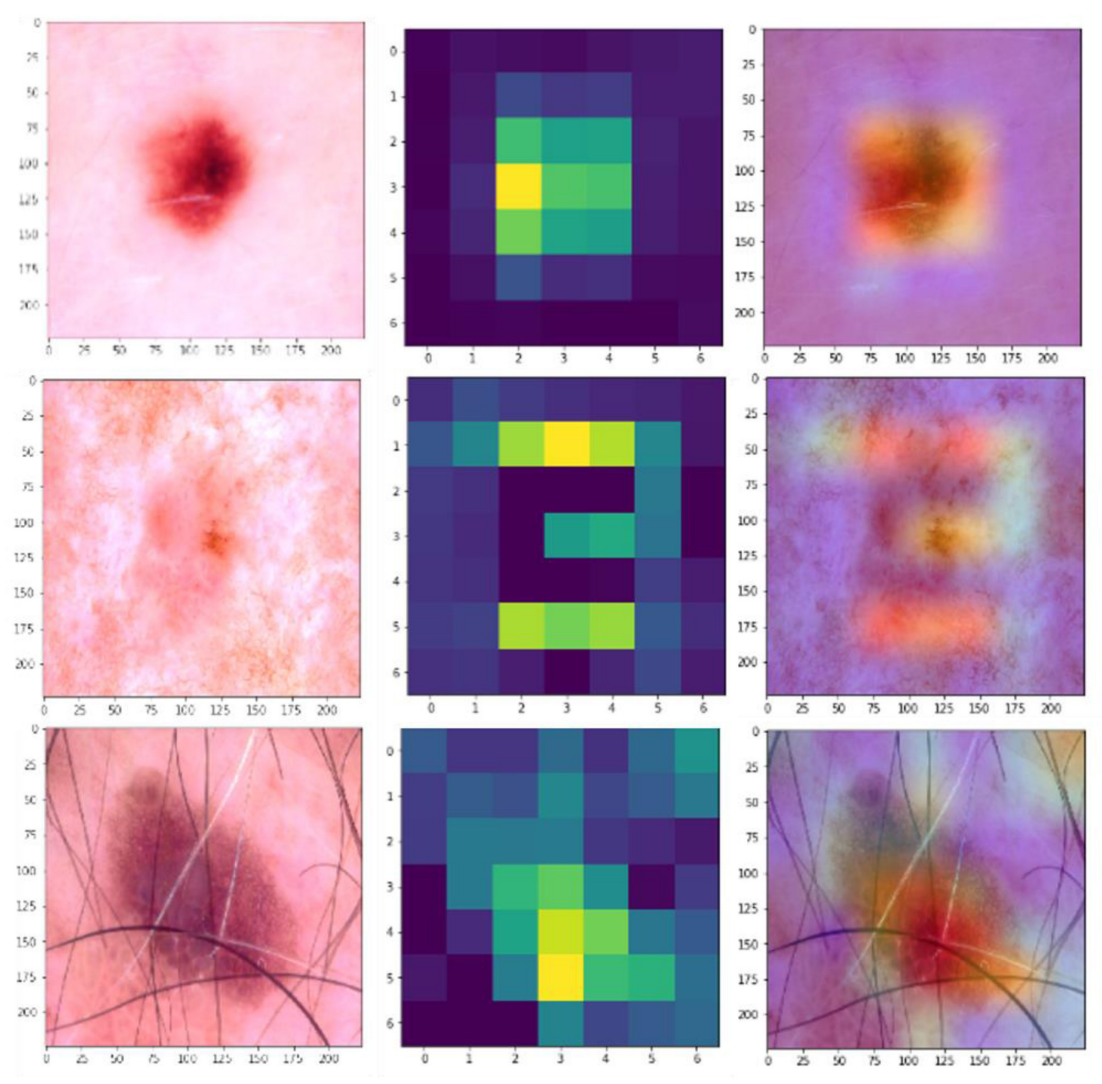
Grad-CAM Visualizations.

Overall, the ablation study underscores the importance of the fusion strategy and the point of integration within the network, with early insertion via simple addition or weighted summation proving to be the most effective in enhancing the model's performance for skin disease classification. To further illustrate the effectiveness of the proposed hybrid model, Gradient-weighted Class Activation Mapping (Grad-CAM) was used to visualize the regions of the dermatological images that the model focuses on when making predictions. Figure 6 shows the Grad-CAM visualizations for a selection of dermatological images from the HAM1000 dataset. Each pair of images consists of the original input image (left), the raw Grad-CAM (middle), and the corresponding overlayed Grad-CAM heatmap (right), which highlights the areas that the model considers important for classification.

Building on the promising results obtained with the HAM10000 datasets, the performance of the proposed hybrid architecture is evaluated on another dataset to validate its efficacy across diverse conditions. ISIC2017 dataset was selected, renowned for its comprehensive collection of skin lesion images, as a new testing ground.

The experimental results, as documented in Table 6, reveal the model's consistent performance. This step was crucial to demonstrate the model's robustness and applicability beyond a single dataset. This cross-dataset validation underscores the model's adaptability and scalability to different medical imaging challenges, marking a significant milestone in the pursuit of advanced diagnostic solutions.

In recent research on skin lesion classification, several approaches have been proposed to improve the accuracy of classification models. Table 7 gives a comparison of various methods in terms of accuracy and F1-score.

In the comparative analysis of the performance of various deep learning models in the field of medical image classification, the proposed hybrid model outperformed many existing approaches. Specifically, it achieved higher accuracy than Sevli et al. 40 and Serti et al. 29, and although the Soft-Attention method 39 had a higher F1-score, the proposed model provided a better overall balance of accuracy and precision.

While it is true that Alenezi's method demonstrates higher accuracy metrics on the HAM10000 and ISIC2017 datasets, the presented approach offers a significant advantage in terms of computational efficiency. The proposed model achieves an execution time of 0.006-0.008 seconds, compared to Alenezi et al.'s 2.3756 seconds 28. This improvement in computational efficiency can be crucial in clinical settings where real-time analysis is required. Additionally, the method's hybrid architecture combining wavelet decomposition with EfficientNet models provides a robust framework that can be further optimized for higher accuracy while maintaining or improving computational efficiency.

### Limitations

While the integration of wavelet transformation with EfficientNet architecture to create a hybrid model for skin disease classification offers promising results, some limitations should be acknowledged. Firstly, the computational complexity introduced by wavelet transformation may increase the overall processing time, potentially limiting real-time applications. In fact, the effectiveness of wavelet transformation can vary depending on the quality and resolution of the input images, potentially impacting the model's robustness and generalizability across diverse datasets. The hybrid approach also requires careful tuning of hyperparameters and integration strategies, which can be challenging and time-consuming. As a result, this approach needs to be re-evaluated when changing the dataset to ensure its effectiveness and adaptability to new data conditions. Finally, while the model aims to generalize across various skin diseases, it may still struggle with rare conditions that are underrepresented in the training data (e.g., minor classes in the HAM1000 dataset like vasc, df, bcc, and akiec).

### Future work and recommendations

The improved accuracy of the proposed hybrid model offers significant clinical benefits, especially in early skin cancer detection, but the added complexity of integrating wavelet transformation with deep learning must be carefully balanced against its marginal performance gains. Future research should focus on optimizing this tradeoff to reduce computational demands, making the model more practical for clinical use. Additionally, fusing the image-based model with tabular data, such as patient demographics or medical history, could enhance diagnostic accuracy and enable personalized treatment recommendations. Improving performance on minority classes remains crucial, with future work exploring adaptive resampling or cost-sensitive learning to better address class imbalance and ensure reliable detection of critical conditions like melanoma.

## Conclusion

The advent of deep learning has revolutionized the field of dermatological medical image analysis, offering unprecedented opportunities for enhancing diagnostic accuracy and patient care. In this study, a hybrid model for the classification of skin diseases is developed by integrating wavelet transformation into the EfficientNet architecture. This innovative approach leverages the strengths of both wavelet transformation, which effectively captures multi-scale features, and EfficientNet, known for its superior performance in image classification tasks. The hybrid model based on the fusion with 2nd-level wavelet decomposition improved accuracy and robustness in distinguishing between various skin diseases, outperforming the baseline EfficientNetB0 model, achieving an accuracy of 94.7% and an F1-score of 94%. Further validation on the ISIC2017 dataset reinforced the model's consistent performance, with an accuracy of 92.2% and an F1-score of 91.9%. These results underscore the robustness and scalability of the proposed hybrid model across different datasets.

Despite the discussed limitations, the findings suggest that the integration of wavelet transformation with advanced neural network architectures holds significant promise for enhancing diagnostic accuracy in dermatology. Future work should focus on expanding the dataset diversity, optimizing computational efficiency, and improving model interpretability to facilitate broader clinical application and acceptance.

## Figures and Tables

**Table 1:** Comparative analysis of methodologies in related studies

Study	Methodology	Datasets	Performance Metrics
Indira et al. 31	Texture Analysis, Multi-level Wavelet Transformation	Not Specified	-
Alenezi et al. 28	WT-DRNNet, ReLU-based ELM, Transfer Learning	ISIC2017, HAM10000	Accuracy: 96.91% (ISIC2017), 95.73% (HAM10000)
Serte et al. 29	Wavelet Coefficients, Deep Learning, Transfer Learning (ResNet-18, ResNet-50)	ISIC2017	M-AUC: 96%, M-ACC: 85%
Chatterjee et al. 30	Empirical Wavelet Transform, EWFD, Ensemble Multiclass Classification	ISIC	Sensitivity: 99.20% (Melanoma), 98.60% (Nevus), 98.20% (BCC), 98.80% (SK)
Aboulmira et al. 32	Fast Fourier Transform, FFT based Convolution	HAM10000	Accuracy: 88%
Proposed Model	Hybrid CNN, Fusion with Wavelet Coefficients	ISIC2017, HAM10000	Accuracy: 92.2% (ISIC2017), 94.7% (HAM10000)

**Table 2:** Performance Comparison of EfficientNet with Different Wavelet Decompositions

Inf. Time	Model	Accuracy	Precision	Recall	F1-score
60 ms/st	Naive Wavelet-CNN model	86%	85%	86%	84%
77 ms/st	EfficientNet backbone	92.5%	93%	92%	91%
82 ms/st	EfficientNet with first wavelet decomposition	93.9%	94%	93%	93%
82 ms/st	EfficientNet with second wavelet decomposition	94.7%	95%	94%	94%
82 ms/st	EfficientNet with third wavelet decomposition	92.5%	93%	92%	91%
82 ms/st	EfficientNet with fourth wavelet decomposition	93.9%	94%	93%	93%
96 ms/st	Fusion EfficientNet with all wavelet decomposition	94.7%	95%	94%	94%

**Table 3:** Per-Class Performance Metrics on HAM10000 Dataset

F1-score			
Methods	Naive Wavelet CNN	Baseline EfficientnetB0	Fusion with 2nd level wavelet decomposition
AKIEC	0.50	0.64	0.78
BCC	0.67	0.82	0.86
BKL	0.68	0.77	0.81
DF	0.60	0.63	0.91
MEL	0.45	0.51	0.77
NV	0.97	0.97	0.98
VASC	0.85	0.81	0.92

**Table 4:** Experimental results of Wave-EfficientNet in terms of accuracy, macro and micro roc

Method	Micro Roc score	Macro Roc score	Accuracy
Proposed approach	0.996	0.988	0.947

**Table 5:** Performance Comparison of Ablation Study Configurations

Configuration	Accuracy	Precision	Recall	F1-score
Early Wavelet Feature Insertion (Block 2A)				
Wavelet Features via Direct Addition (Add layer)	94.3%	94%	94%	94%
Wavelet Features via Concatenation Followed by 1x1 Convolution	94.13%	94%	95%	95%
Fusion via Weighted Summation	94.7%	95%	94%	94%
Fusion via Attention Mechanism	93.60%	93%	94%	93%
Late Wavelet Feature Fusion (with final EfficientNet layer)				
Wavelet Features via Direct Addition	93.92%	93%	94%	93%
Wavelet Features via Concatenation Followed by 1x1 Convolution	91.79%	92%	92%	92%

**Table 6:** Comparison of overall performance: accuracy, precision, recall and F-1 score for Isic2017

Method	Accuracy	Precision	Recall	F1-score
Proposed approach	92.2%	92%	91.8%	91.9%

**Table 7:** Comparison of Skin Lesion Classification Methods in Terms of Accuracy and F1-Score

Method	Dataset	Accuracy	F1-score
Proposed approach	HAM10000	94.7%	95%
	ISIC2017	92.2%	91.9%
			
WT-DRNNet 28	HAM10000	96.91%	95.79%
	ISIC2017	95.73%	93.44%
Soft-Attention 39	HAM10000	93.4%	98.4%
Custom CNN architecture 40	HAM1000	91.51%	-
ResNet-50-18 with I-A1-A2-A3 29	ISIC2017	85%	-
